# Effects of genetic deficiency of cyclooxygenase-1 or cyclooxygenase-2 on functional and histological outcomes following traumatic brain injury in mice

**DOI:** 10.1186/1471-2202-10-108

**Published:** 2009-08-31

**Authors:** Matthew L Kelso, Stephen W Scheff, James R Pauly, Charles D Loftin

**Affiliations:** 1 Department of Pharmaceutical Sciences, College of Pharmacy, University of Kentucky, Lexington, KY, USA; 2 Sanders-Brown Center on Aging, University of Kentucky, Lexington, KY, USA; 3 Spinal Cord and Brain Injury Research Center, University of Kentucky, Lexington, KY, USA

## Abstract

**Background:**

Neuroinflammation contributes to the pathophysiology of acute CNS injury, including traumatic brain injury (TBI). Although prostaglandin lipid mediators of inflammation contribute to a variety of inflammatory responses, their importance in neuroinflammation is not clear. There are conflicting reports as to the efficacy of inhibiting the enzymes required for prostaglandin formation, cyclooxygenase (COX) -1 and COX-2, for improving outcomes following TBI. The purpose of the current study was to determine the role of the COX isoforms in contributing to pathological processes resulting from TBI by utilizing mice deficient in COX-1 or COX-2.

**Results:**

Following a mild controlled cortical impact injury, the amount of cortical tissue loss, the level of microglial activation, and the capacity for functional recovery was compared between COX-1-deficient mice or COX-2-deficient mice, and their matching wild-type controls. The deficiency of COX-2 resulted in a minor (6%), although statistically significant, increase in the sparing of cortical tissue following TBI. The deficiency of COX-1 resulted in no detectable effect on cortical tissue loss following TBI. As determined by ^3^[H]-PK11195 autoradiography, TBI produced a similar increase in microglial activation in multiple brain regions of both COX-1 wild-type and COX-1-deficient mice. In COX-2 wild-type and COX-2-deficient mice, TBI increased ^3^[H]-PK11195 binding in all brain regions that were analyzed. Following injury, ^3^[H]-PK11195 binding in the dentate gyrus and CA1 region of the hippocampus was greater in COX-2-deficient mice, as compared to COX-2 wild-type mice. Cognitive assessment was performed in the wild-type, COX-1-deficient and COX-2-deficient mice following 4 days of recovery from TBI. There was no significant cognitive effect that resulted from the deficiency of either COX-1 or COX-2, as determined by acquisition and spatial memory retention testing in a Morris water maze.

**Conclusion:**

These findings suggest that the deficiency of neither COX-1 nor COX-2 is sufficient to alter cognitive outcomes following TBI in mice.

## Background

The cyclooxygenases (COX-1 and COX-2) are key modulators of inflammation because they are required for the synthesis of prostaglandin (PG) H_2_, the substrate utilized for the synthesis of all biologically active PGs and thromboxane A_2 _[1]. Although the activity of both COX-1 and COX-2 results in the production of the same initial product (PGH_2_), there are significant differences in the expression characteristics of the two enzymes. Originally, COX-1 was described as a "housekeeping" enzyme because of constitutive expression in a variety of peripheral tissues, whereas COX-2 expression was not detected under physiological conditions but was induced following pathogenic challenge.

Although there are a limited number of studies examining the function of COX-1 expressed in the brain, it is clear that the pattern of COX-1 expression in the CNS is distinct from the constitutive expression observed in peripheral tissues. In rats, COX-1 expression is significantly increased by traumatic brain injury (TBI) with the increased expression being localized to accumulating cells within and adjacent to the developing injury [2]. The majority of the cells expressing COX-1 also express markers of activated microglia, and the increased COX-1 expression begins during the first day following the injury [2]. Similarly in humans, TBI produces increased accumulation of COX-1 expressing activated microglia that localize at the site of injury [3]. The increased accumulation of COX-1 expressing microglia has also been identified in other types of CNS injury, including ischemic injury [4,5], and pharmacological inhibition of COX-1 has been shown to be effective in reducing neuronal damage following ischemia [6]. These studies showing increased expression and a potential causative role for COX-1 have suggested that COX-1 inhibition may provide a more effective therapy for TBI than inhibition of COX-2 [2].

There are a considerable number of reports examining the expression pattern or function of COX-2 in the brain. In addition to being constitutively expressed [7-9] and providing physiological functions in the mammalian CNS [10], increased COX-2 expression also accompanies numerous types of CNS injury. COX-2 expression is rapidly induced in the cortex and hippocampus following experimental TBI [11,12]. COX-2 has been implicated in the permeability of the blood-brain barrier (BBB) and CNS infiltration of circulating leukocytes [13]. COX-2 may also promote brain edema in experimental intracerebral hemorrhage [14], though this role in TBI models has been questioned [15]. Despite the finding that COX-2-dependent PGs propagate microglial activation [16], the utility of COX-2 inhibition following TBI has been debated as several studies have shown no benefit using this strategy [15,17,18]. In addition, a recent report using genetically deficient mice, showed no effect of COX-2 deficiency on histological or behavioral outcomes following TBI [19]. With the conflicting reports on the roles of the two COX isoforms, we examined the effects of TBI in mice deficient in either COX-1 or COX-2.

## Methods

### Animals

The current study included 29 COX-2 +/+, 24 COX-2 -/- adult (2 - 6 month), and 10 COX-1 +/+, and 10 COX-1 -/- adult (2 month), male mice bred on a C57BL/6 × 129/Ola (C57/129) background that were obtained from Taconic Farms Inc. [20,21]. These mice and their matching wild-type littermate controls were produced by crossing COX-1 heterozygous mice or COX-2 heterozygous mice that have been maintained for more than 35 generations. The mice were housed 2 - 4 per cage in a temperature-controlled environment on a 12-hour light/dark cycle. Animals were given free access to food and water and all experimental procedures were reviewed and approved by the Institutional Animal Care and Use Committee at the University of Kentucky and carried out in accordance with the National Institute of Health Guide for the Care and Use of Laboratory Animals. All efforts were made to minimize pain or discomfort.

### Controlled Cortical Impact (CCI)

The mice were subjected to a cortical contusion injury as described previously [22,23]. COX-1 +/+ and -/- mice were anesthetized with intraperitoneal (i.p.) tribromoethanol (Avertin 0.175 mg/kg, Aldrich, St. Louis, MO). Because a significant level (63%) of mortality occurred in COX-2 -/- mice following i.p. tribromoethanol, COX-2 +/+ and -/- mice were anesthetized with 2% inhaled isoflurane. After securing in a Kopf stereotaxic frame (David Kopf, Tujunga, CA), a midline incision was performed and the scalp was reflected so that a 4 mm craniotomy could be performed midway between bregma and lambda. Care was taken during this procedure to expose the somatosensory cortex without disturbing the dura mater. The frame was positioned under a TBI 0310 impaction device (Precision Systems & Instrumentation, Fairfax Station, VA) that was set to deliver a mild impact (0.3 mm impact depth, 3 mm tip diameter, 3.5 m/s velocity, 400 msec dwell time) to the cortical surface. Following impact, Surgicel (Johnson & Johnson) was placed over the craniotomy and the incision was stapled closed. The core body temperature of the animals was 36 - 37°C throughout the procedure. The mice were then returned to their home cages and allowed to recover for 4 days before further experimentation.

### Baseline Cognitive Assessment

In preliminary experiments, the COX-1 and COX-2 wild-type and null mutant animals were trained in the Morris water maze (MWM) prior to surgery to evaluate possible genotypic differences in baseline spatial learning ability. Briefly, animals were tested in a 127 cm diameter × 56 cm tall circular pool with a 13.5 cm diameter circular platform submerged approximately 1 cm below the waterline and located in the middle of the SE quadrant. White, non-toxic powdered paint was added to the water in order to obscure the platform. The lighting of the room and various spatial cues were constant throughout the training period. The animals were given 4 - 60 second trials/day with their entry points (N, S, E, W) being pseudo-randomized each day. If at the end of the 60 sec trial the animal did not find the platform, the animal was placed there by the handler and allowed to rest for 15 sec. The animals were allowed a 5-minute rest between each trial. The animals were trained daily until they reached asymptote, which was reached for each group after 4 days.

### Post-Injury Cognitive Assessment

COX-2 inhibition has been shown to significantly impair motor function when analyzed from 1 to 3 days following CCI injury in rats [24]. However, the adverse effect on motor function was not observed by the 4-day time-point [24]. To avoid potential effects on motor function, the current study analyzed the cognitive effects of either COX-1 or COX-2 deficiency using the Morris water maze (MWM) 4 days following TBI. Following the last trial on the 5^th ^day, the animals were allowed a 1-hour rest period before performing the retention trial where the platform was removed and the animals were placed in the pool at a unique location (NW) and given 30 seconds to explore the pool. The performance in the MWM was analyzed using a video motion analyzer (Videomex V, Columbus Instruments, Columbus, OH).

### Tissue Sparing Analysis

Following the memory retention test, the animals were euthanatized by cervical dislocation, their brains rapidly removed and flash frozen on finely sifted dry ice. The brains were kept at -80°C until processing. The frozen tissue was sectioned to 16 μm thick coronal slices on a Leica 1850 M cryostat (Nussloch, Germany) and eight equally spaced sections/slide were thaw mounted onto Fisher SuperFrost Plus^® ^slides. Serial sets of slides were collected so that multiple assays could be conducted at similar anatomical levels, which resulted in sections that were approximately 128 μm apart on each slide. The slides were stored under vacuum overnight at 4°C then transferred to -80°C until time of experimentation.

To assess the potential neuroprotective effect of COX deletion from loss of cortical parenchyma, a tissue sparing analysis was performed as previously described [23]. One set of slides was removed from -80°C and allowed to thaw overnight. The slides were then stained with cresyl violet and coverslipped. Images of the slices between Bregma -0.94 mm and -2.54 mm [25] were obtained and the mean cortical area, defined as the area between lamina 1 and the corpus callosum, was quantified using ImageJ software (NIH, Bethesda, MD). The mean cortical area of the cortex ipsilateral to the impact was divided by the mean cortical area of the corresponding contralateral cortex (× 100) to determine the percentage of tissue spared. Utilization of this method allows that each animal can serve as its own control and accounts for changes to the tissue that may occur during processing [26]. The animals were not identified by genotype until all measurements had been completed.

### ^3^[H]-PK11195 Autoradiography

Microglial activation was assessed by detection of ^3^[H]-PK11195 binding to the translocator protein (TSPO) as described previously [23,27]. Slides were removed from -80°C and allowed to thaw overnight at room temperature, loaded into binding racks and incubated in 50 mM Tris HCl buffer (pH 7.4) for 15 minutes at 4°C. The racks were then transferred to incubation buffer containing 50 mM Tris HCl and 1 nM [^3^H]-PK11195 (PerkinElmer, Boston, MA, specific activity = 73.6 Ci/mmol). The slides incubated for 2 hours at 4°C followed by 3 washes in 50 mM Tris HCl buffer (pH 7.4) at 4°C for 3 minutes each and a brief wash in ddH_2_O at 4°C. The slides were left overnight to dry at room temperature and were then placed into Fisher Biotech Autoradiography Cassettes and exposed to Kodak BioMax film for approximately 5 weeks. Following exposure, the film was developed using Kodak GBX developer. The brain regions analyzed were selected based on previous studies that have shown increased microglia activation in these regions following brain injury [23,27,28].

### Statistical Methods

MWM performance data were analyzed by 2-way (genotype × day), repeated measures (day) ANOVA. Group comparisons between the COX-1 +/+ and -/- populations and the COX-2 +/+ and -/- populations for performance in the target quadrant during the probe trial were analyzed by t-test. Tissue sparing data for two groups was also analyzed by t-test. [^3^H]-PK11195 autoradiography results were analyzed by 2-way (genotype × hemisphere) repeated measures (hemisphere) ANOVA. All post-hoc tests were conducted using Student Newman-Keuls. Significance was defined as p < 0.05. Data are represented as mean ± SD.

## Results

### Baseline Cognitive Assessment

**Figure 1:**
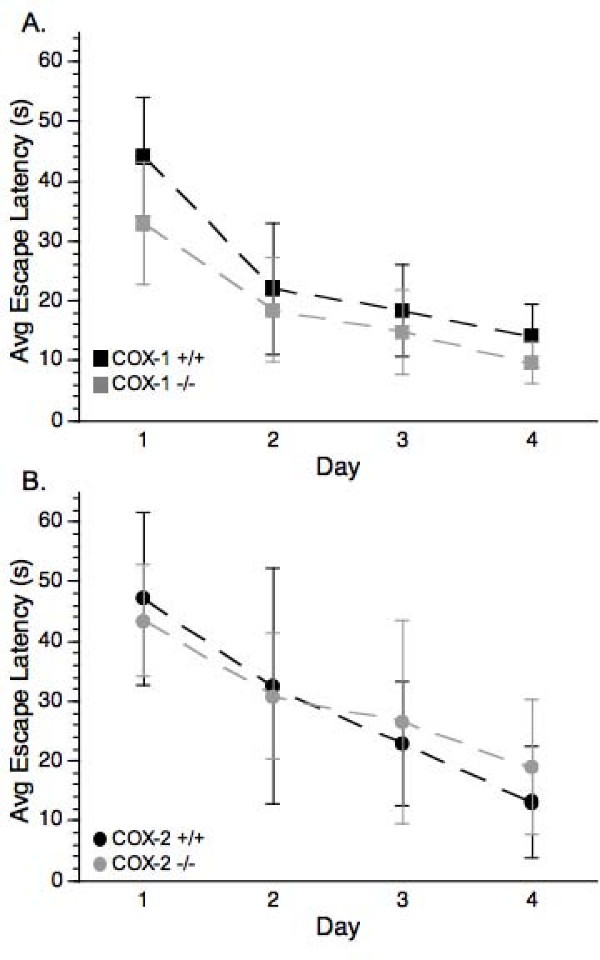
**Assessment of pre-injury spatial learning ability as determined by the MWM**. (A) COX-1 +/+ and COX-1 -/- mice or (B) COX-2 +/+ and COX-2 -/- mice showed a significant day effect (ANOVA *P *< 0.05) indicating that each genotype was able to learn the location of the hidden platform. Data are presented as mean ± SD.

Analysis of the MWM escape latency in the pre-injury COX-1 +/+ and COX-1 -/- mice revealed that there was a significant day effect [F(3, 54) = 43.40, p < 0.0001] but there was no effect of genotype, or any genotype X day interaction (Figure 1A). Analysis of the pre-injury performance of the COX-2 +/+ and COX-2 -/- animals revealed a significant day effect [F(3,51) = 0.81, p < 0.0001] that followed a similar pattern as described for the COX-1 animals (Figure 1B), where there were no differences between the abilities of the wild-type and null mutant mice to learn the location of the platform before injury. There was also no significant difference between the sham-operated wild-type and null mice in any measure of the MWM (data not shown). These findings indicate that prior to injury neither the deficiency of COX-1 nor COX-2 significantly affected cognitive ability.

### Post-Injury Cognitive Assessment

**Figure 2:**
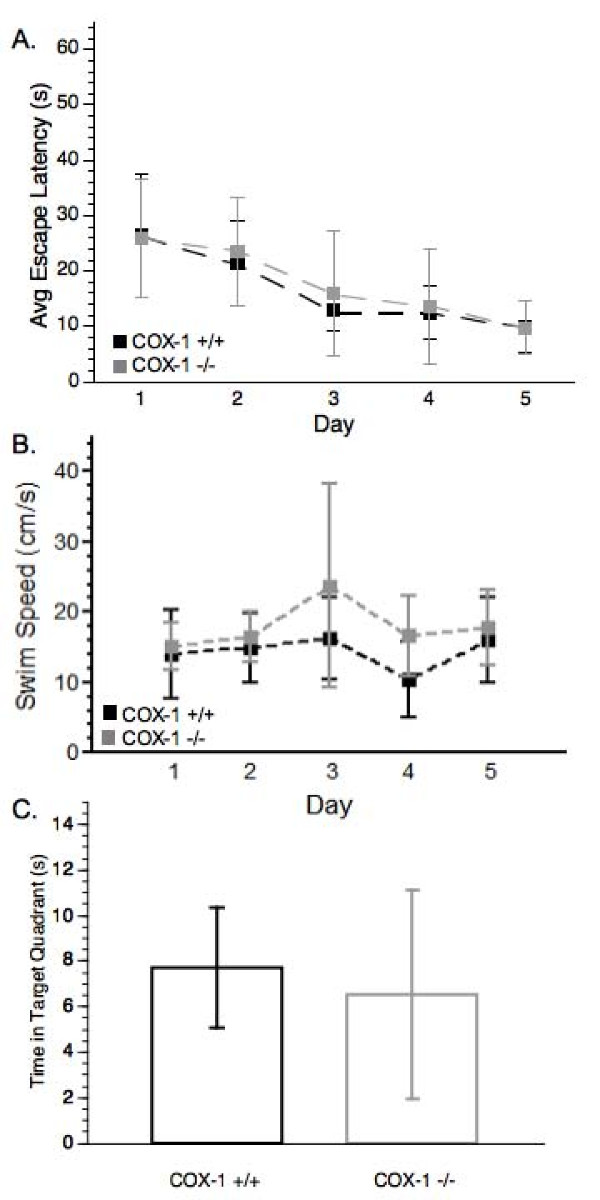
**Post-injury cognitive assessment and probe trial of COX-1 +/+ and COX-1 -/- mice**. (A) Post-injury MWM escape latency. There was a significant effect of day (ANOVA *P *< 0.0001) for both genotypes, but there was no significant difference between COX-1 +/+ and COX-1 -/- mice. (B) Analysis of swim speed during the probe trial of the MWM. (C) Duration of time mice remained in the target quadrant during the probe trial of the MWM. Data are presented as mean ± SD.

Following TBI, average latency increased from 14.18 ± 5.29 sec and 9.6 ± 3.54 sec, for COX-1 +/+ and -/- animals, respectively, to 26.44 ± 11.06 sec and 25.97 ± 10.76 sec, suggesting that CCI impaired the ability of these animals to remember the location of the submerged platform. For both groups over the course of the 5-day post-injury testing period, there was a significant day effect for escape latency [F(4, 64) = 18.48, p < 0.0001] with both COX-1 +/+ and COX-1 -/- mice being equally capable of learning the task, and no significant differences between the genotypes (Figure 2A). There was also no significant difference in swim speed between COX-1 +/+ mice and COX-1 -/- mice (Figure 2B). During the probe trial, COX-1 +/+ and COX-1 -/- mice did not show a significant difference in distance traveled in the target quadrant [t(16) = 0.59, p > 0.05] (Figure 2C) suggesting that there was no effect of COX-1 gene deletion on spatial navigation capabilities following TBI.

**Figure 3:**
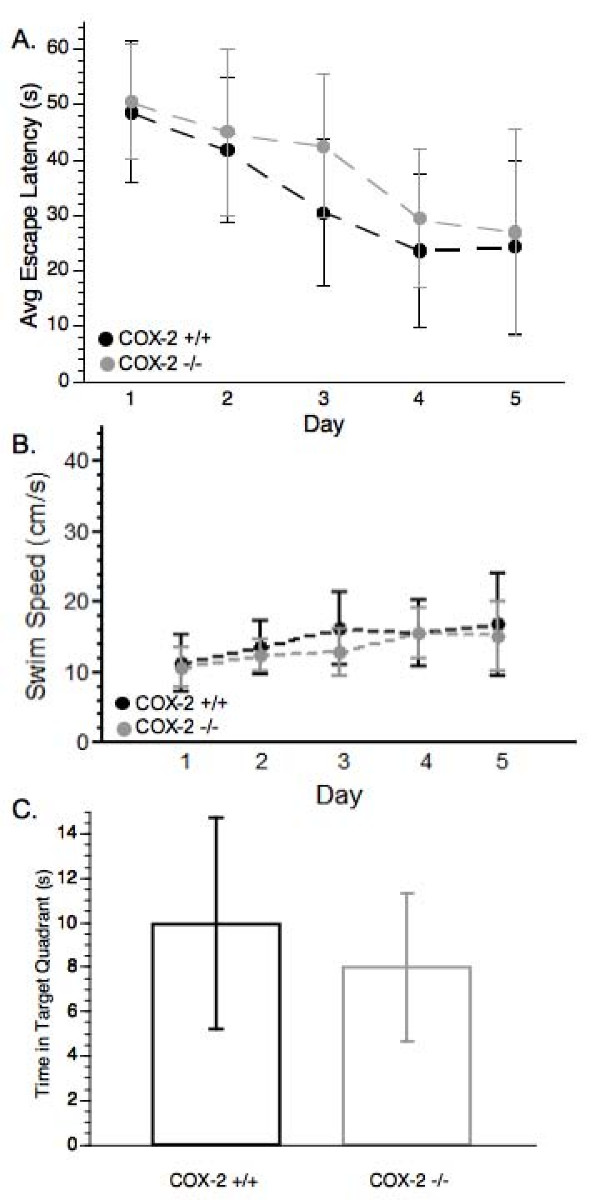
**Post-injury cognitive assessment and probe trial of COX-2 +/+ and COX-2 -/- mice**. (A) Post-injury MWM acquisition latency. COX-2 +/+ and COX-2 -/- mice showed a significant day effect (*P *< 0.0001) but no significant differences between the two genotypes. (B) Analysis of swim speed during the probe trial of the MWM. (C) Duration of time in the target quadrant during the probe trial of the MWM. Data are presented as mean ± SD.

The average latency following TBI increased from 13.13 ± 9.42 sec to 25.08 ± 10.72 sec for the COX-2 +/+ mice and from 18.91 ± 11.26 sec to 29.67 ± 9.40 sec for the COX-2 -/- mice. These findings of increased latency suggest a similar adverse effect of the injury on both genotypes ability to remember the location of the submerged platform. COX-2 +/+ and -/- animals were able to learn the MWM task as indicated by significant decreases in latency time [F(4, 132) = 29.38, p < 0.0001] (Figure 3A) during the post-injury testing phase, however there were no differences between the groups. COX-2 wild-type and null mutant animals also did not differ in either swim speed (Figure 3B) during the 5 day acquisition testing or the length of time in the target quadrant during the probe trial (Figure 3C). Taken together, these data suggest that neither COX-1 nor COX-2 gene deletion is a critical factor in altered spatial memory function following TBI.

### Tissue Sparing Analysis

**Figure 4:**
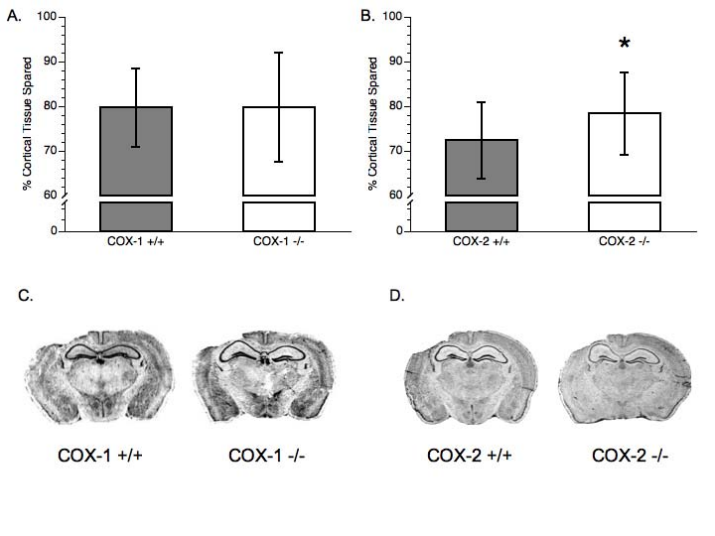
**Tissue sparing analysis**. (A) Comparison of cortical tissue loss between COX-1 +/+ mice and COX-1 -/- mice. (B) Comparison of cortical tissue loss between COX-2 +/+ mice and COX-2 -/- mice. (C) Pictorial representation of cortical tissue sparing following CCI in COX-1 +/+ and COX-1 -/- mice. (D) Pictorial representation of cortical tissue sparing following CCI in COX-2 +/+ and COX-2 -/- mice. (*) Represents significance from wild-type controls (*P *< 0.05). Data presented as mean ± SD.

There was no statistically significant difference in the amount of cortical tissue spared between the COX-1 +/+ and COX-1 -/- groups [t(12) = 0.01, p > 0.05] (Figure 4A, C). There was a statistically significant effect on cortical tissue sparing that resulted from the deficiency of COX-2 [t(36) = -2.07, p < 0.05]. COX-2 +/+ animals had approximately 72.3 ± 8.6% of their cortical tissue spared compared, as compared to 78.3 ± 9.2% in the COX-2 -/- animals (Figure 4B, D).

### ^3^[H]-PK11195 Autoradiography

In COX-1 +/+ and COX-1 -/- mice, results from a two-way, repeated measures ANOVA showed a significant increase in [^3^H]-PK11195 binding in the injured hemisphere compared to the uninjured hemisphere for the majority of the brain regions analyzed (Table 1). The [^3^H]-PK11195 binding following injury was not significantly increased in the cortex of both genotypes, or the CA1 region of COX-1 +/+ mice. The CA1 region showed a genotypic effect as the COX-1 -/- animals produced less [^3^H]-PK11195 binding in both the injured and uninjured hemisphere compared to the COX-1 +/+ animals [F(1,13) = 18.61, p < 0.001], suggesting less microglial activation.

**Table 1:** Binding of [^3^H]-PK11195 following cortical contusion injury in COX-1 +/+ and COX-1 -/- mice.

	COX-1 +/+		COX-1 -/-	
	
Brain Region	Uninjured	Injured	Uninjured	Injured
CA1	5.35 ± 0.97	5.55 ± 1.07	3.71 ± 0.50^†^	4.46 ± 0.38*^†^
CTX	3.01 ± 0.23	3.63 ± 0.70	2.95 ± 0.22	3.36 ± 0.35
DLG	2.65 ± 0.18	4.09 ± 0.79*	2.48 ± 0.14	4.15 ± 1.10*
LPM	2.83 ± 0.15	3.72 ± 0.41*	2.66 ± 0.26	3.87 ± 0.85*
DG	5.00 ± 1.58	7.15 ± 2.52*	4.66 ± 0.54	6.59 ± 0.78*
VPL/VPM	2.16 ± 0.14	2.56 ± 0.27*	2.07 ± 0.18	2.54 ± 0.40*

Binding studies conducted on mice euthanatized 9 days after injury. Data is expressed as mean ± SD of μCi/mg of wet tissue. * represents significant changes compared to the uninjured hemispere; † represents significant changes compared to the corresponding hemisphere of the COX-1 +/+ animals. CA1, CA1 region of the hippocampus; CTX, cortex; DLG, dorsal lateral geniculate nucleus; LPM, lateral posterior nucleus; DG, dentate gyrus; VPL/VPM, ventral posteriolateral/medial thalamic nucleus.

A significant increase in [^3^H]-PK11195 binding was observed in all brain regions of the injured hemisphere for both the COX-2 +/+ and COX-2 -/- animals (Table 2). In addition to the main effect of hemisphere, there was a significant interaction of genotype X hemisphere in the CA1 region [F(1,36) = 9.27, p < 0.01] and the dentate gyrus [F(1,37) = 5.08, p < 0.05]. For both of these regions, post-hoc analysis showed a significant increase in [^3^H]-PK11195 binding in the injured hemisphere of COX-2 -/- mice, as compared to the injured hemisphere of COX-2 +/+ mice. To exclude the possibility that the increases in [^3^H]-PK11195 binding in the hippocampus were due to differences between genotypes prior to injury, a binding assay was performed on sham operated COX-2 +/+ and COX-2 -/- animals. There was no difference in [^3^H]-PK11195 binding between these two groups (data not shown).

**Table 2:** Binding of [^3^H]-PK11195 following cortical contusion injury in COX-2 +/+ and COX-2 -/- mice.

	COX-2 +/+		COX-2 -/-	
	
Brain Region	Uninjured	Injured	Uninjured	Injured
CA1	3.22 ± 0.82	3.88 ± 0.47*	2.97 ± 0.61	4.89 ± 1.43*†
CTX	2.72 ± 0.43	4.05 ± 0.87*	2.77 ± 0.42	4.09 ± 0.70*
DLG	2.28 ± 0.20	3.36 ± 0.84*	2.24 ± 0.31	3.40 ± 0.66*
LPM	2.51 ± 0.23	3.40 ± 0.62*	2.52 ± 0.30	3.68 ± 0.92*
DG	4.66 ± 1.17	5.87 ± 0.80*	4.36 ± 0.96	6.63 ± 1.59*†
VPL/VPM	2.02 ± 0.14	2.41 ± 0.20*	1.97 ± 0.25	2.45 ± 0.41*

Binding studies conducted on mice euthanatized 9 days after injury. Data is expressed as mean ± SD of μCi/mg of wet tissue. * represents significant changes compared to the uninjured hemisphere; † represents significant changes compared to the corresponding hemisphere of the COX-2 +/+ animals. CA1, CA1 region of the hippocampus; CTX, cortex; DLG, dorsal lateral geniculate nucleus; LPM, lateral posterior nucleus; DG, dentate gyrus; VPL/VPM, ventral posteriolateral/medial thalamic nucleus.

## Discussion

The majority of reports examining contributions of the two COX isoforms to neuroinflammation have focused on a causative role for the COX-2 isoform. Much of the emphasis on COX-2 may result from the availability and therapeutic success of COX-2-selective inhibitors. However, studies utilizing COX-1-deficient [29] or COX-2-deficient [30] mice have shown that inactivation of COX-1 reduces neuroinflammation whereas COX-2 inactivation worsens the neuroinflammatory response. Furthermore, in both humans and animal models, TBI results in the prolonged accumulation of COX-1 expressing microglia in the region of the developing lesion [2,3]. Therefore, findings from previous reports would support the strategy of COX-1 inactivation as a potential method for improving outcomes following TBI. However, as described in the current study using the MWM method of determining cognitive functioning, there was no identifiable effect of COX-1 deficiency on behavioral outcomes following TBI in mice.

The strategy of using pharmacological inhibition of COX-2 for amelioration of behavioral outcomes resulting from TBI has produced differing results. In rats, COX-2 inhibition has been shown to improve cognitive functioning as determined by the MWM following a severe 3 mm CCI [31] and by the Barnes maze following an impact-acceleration model of diffuse TBI [32]. However, Dash et al. [24] did not observe any differences in MWM performance between administration of the COX-2 inhibitor celecoxib or vehicle, following a milder 2 mm CCI. Another experimental brain injury study failed to see an improvement in the MWM following COX-2 inhibitor administration to juvenile rats [17]. When the weight drop model was used to induce brain injury, the COX-2 inhibitor nimesulide did not improve Neurological Severity Score (NSS) [15]. Furthermore, the MWM was recently used as an outcome measure following CCI performed on COX-2 null mutant mice. As we observed in our current study, Ahmed et al. found no significant cognitive effect resulting from COX-2 gene deletion following a 1.2 mm CCI in mice [19].

In addition to the behavioral results discussed above, there have also been inconsistent findings with studies using biochemical and histological endpoints to examine the effectiveness of COX-2 inhibition following TBI. Gopez et al. [31] showed that COX-2 inhibition decreased neuronal expression of the apoptotic marker, activated caspase-3, and reduced PGE_2 _levels in the brain. Hickey et al. [17] also found that COX-2 inhibition decreased PGE_2 _production, but this decrease was not accompanied by decreased brain edema or increased tissue sparing, an effect also seen by Koyfman et al. [15]. Further investigation on neuroprotective effects of COX-2 inhibition up to 72 hours following injury showed no significant change in degenerating neuronal cell bodies, as detected with fluoro-jade B, or in DNA fragmentation, as determined by TUNEL staining [18]. In contrast, COX-2 null mutants subjected to a 1.2 mm CCI did show a significant decrease in TUNEL positive cells at 24 hours after injury [19]. In our current study, we observed a 6% difference in the amount of cortical tissue spared between the COX-2 +/+ mice and COX-2 -/- mice. However, cortical tissue surrounding the epicenter of the cortical impact is heavily damaged, both in cell bodies and axonal processes [22,33]. Because we did not identify improvements in cognitive outcomes resulting from the deficiency of COX-2, the biological significance of the observed 6% improvement in cortical tissue sparing in COX-2-deficient mice is currently not clear.

Recently, there have been conflicting reports as to the specificity of [^3^H]-PK11195 for labeling activated microglia. Multiple neurodegeneration studies have shown a strong correlation between *ex vivo *binding of racemic or the (R) enantiomer of [^3^H]-PK11195 and immunohistochemical markers of microglia [27,28,34-36]. Some of these studies have also shown a correlation between [^3^H]-PK11195 binding and immunohistochemical markers of reactive astrocytes, though the correlation coefficient is much lower than with activated microglia [25,27]. Additionally, [^3^H]-PK11195 binding sites have been found on neutrophils [37], although these cell types are significantly decreased or undetectable by seven days post-injury [38-41]. Therefore, although the increase in [^3^H]-PK11195 binding following brain injury is most often associated with activated microglia, the contribution of other cell types cannot be excluded. Recently, the brains of COX-2-deficient mice were shown to exhibit increased activation of both microglia and astrocytes following lipopolysaccharide administration [30]. Thus, the increased [^3^H]-PK11195 binding that we observed in COX-2-deficient mice may indicate an increased inflammatory response involving multiple cell types.

## Conclusion

In conclusion, we could identify no significant cognitive effect that resulted from the deficiency of either COX-1 or COX-2 following TBI in mice. COX-2 gene deletion produced minor (6%) protection from progressive cavitation while at the same time there was increased microglia activation in the hippocampus. The lack of consensus regarding the benefits or limitations of inhibiting COX-1 or COX-2 for treatment of TBI warrants further research to examine the potential of this strategy.

## Abbreviations

ANOVA: analysis of variance; BBB: blood-brain barrier; CA1: CA1 layer of the hippocampus; CCI: controlled cortical impact; COX: cyclooxygenase; CTX: cortex; DG: dentate gyrus; DLGN: dorsal lateral geniculate nucleus; i.p.: intraperitoneal; LFP: lateral fluid percussion; LPM: lateral posterior nucleus; Mol: molecular layer of the dentate gyrus; MWM: Morris water maze; PG: prostaglandin; SD: standard deviation; TBI: traumatic brain injury; TSPO: translocator protein (18 kDa); VPM/VPL: ventral posteriomedial/lateral thalamic nucleus.

## Authors' contributions

M.L.K. performed the experiments and contributed to manuscript preparation. S.W.S., J.R.P. and C.D.L. coordinated and guided the experimental plans and contributed to manuscript preparation. The final manuscript has been read and approved by all authors.

## References

[B1] Simmons DL, Botting RM, Hla T (2004). Cyclooxygenase isozymes: the biology of prostaglandin synthesis and inhibition. Pharmacol Rev.

[B2] Schwab JM, Seid K, Schluesener HJ (2001). Traumatic brain injury induces prolonged accumulation of cyclooxygenase-1 expressing microglia/brain macrophages in rats. J Neurotrauma.

[B3] Schwab JM, Beschorner R, Meyermann R, Gozalan F, Schluesener HJ (2002). Persistent accumulation of cyclooxygenase-1-expressing microglial cells and macrophages and transient upregulation by endothelium in human brain injury. J Neurosurg.

[B4] Schwab JM, Brechtel K, Nguyen TD, Schluesener HJ (2000). Persistent accumulation of cyclooxygenase-1 (COX-1) expressing microglia/macrophages and upregulation by endothelium following spinal cord injury. J Neuroimmunol.

[B5] Schwab JM, Nguyen TD, Postler E, Meyermann R, Schluesener HJ (2000). Selective accumulation of cyclooxygenase-1-expressing microglial cells/macrophages in lesions of human focal cerebral ischemia. Acta Neuropathol.

[B6] Candelario-Jalil E, Gonzalez-Falcon A, Garcia-Cabrera M, Alvarez D, Al-Dalain S, Martinez G, Leon OS, Springer JE (2003). Assessment of the relative contribution of COX-1 and COX-2 isoforms to ischemia-induced oxidative damage and neurodegeneration following transient global cerebral ischemia. J Neurochem.

[B7] Breder CD, Dewitt D, Kraig RP (1995). Characterization of inducible cyclooxygenase in rat brain. J Comp Neurol.

[B8] Kaufmann WE, Worley PF, Pegg J, Bremer M, Isakson P (1996). COX-2, a synaptically induced enzyme, is expressed by excitatory neurons at postsynaptic sites in rat cerebral cortex. Proc Natl Acad Sci USA.

[B9] Yamagata K, Andreasson KI, Kaufmann WE, Barnes CA, Worley PF (1993). Expression of a mitogen-inducible cyclooxygenase in brain neurons: regulation by synaptic activity and glucocorticoids. Neuron.

[B10] Chen C, Magee JC, Bazan NG (2002). Cyclooxygenase-2 regulates prostaglandin E2 signaling in hippocampal long-term synaptic plasticity. J Neurophysiol.

[B11] Cernak I, O'Connor C, Vink R (2001). Activation of cyclo-oxygenase-2 contributes to motor and cognitive dysfunction following diffuse traumatic brain injury in rats. Clin Exp Pharmacol Physiol.

[B12] Strauss KI, Barbe MF, Marshall RM, Raghupathi R, Mehta S, Narayan RK (2000). Prolonged cyclooxygenase-2 induction in neurons and glia following traumatic brain injury in the rat. J Neurotrauma.

[B13] Candelario-Jalil E, Gonzalez-Falcon A, Garcia-Cabrera M, Leon OS, Fiebich BL (2007). Post-ischaemic treatment with the cyclooxygenase-2 inhibitor nimesulide reduces blood-brain barrier disruption and leukocyte infiltration following transient focal cerebral ischaemia in rats. J Neurochem.

[B14] Chu K, Jeong SW, Jung KH, Han SY, Lee ST, Kim M, Roh JK (2004). Celecoxib induces functional recovery after intracerebral hemorrhage with reduction of brain edema and perihematomal cell death. J Cereb Blood Flow Metab.

[B15] Koyfman L, Kaplanski J, Artru AA, Talmor D, Rubin M, Shapira Y (2000). Inhibition of cyclooxygenase 2 by nimesulide decreases prostaglandin E2 formation but does not alter brain edema or clinical recovery after closed head injury in rats. J Neurosurg Anesthesiol.

[B16] Vijitruth R, Liu M, Choi DY, Nguyen XV, Hunter RL, Bing G (2006). Cyclooxygenase-2 mediates microglial activation and secondary dopaminergic cell death in the mouse MPTP model of Parkinson's disease. J Neuroinflammation.

[B17] Hickey RW, Adelson PD, Johnnides MJ, Davis DS, Yu Z, Rose ME, Chang YF, Graham SH (2007). Cyclooxygenase-2 activity following traumatic brain injury in the developing rat. Pediatr Res.

[B18] Kunz T, Marklund N, Hillered L, Oliw EH (2006). Effects of the selective cyclooxygenase-2 inhibitor rofecoxib on cell death following traumatic brain injury in the rat. Restor Neurol Neurosci.

[B19] Ahmad M, Rose ME, Vagni V, Griffith RP, Dixon CE, Kochanek PM, Hickey RW, Graham SH (2008). Genetic disruption of cyclooxygenase-2 does not improve histological or behavioral outcome after traumatic brain injury in mice. J Neurosci Res.

[B20] Langenbach R, Morham SG, Tiano HF, Loftin CD, Ghanayem BI, Chulada PC, Mahler JF, Lee CA, Goulding EH, Kluckman KD (1995). Prostaglandin synthase 1 gene disruption in mice reduces arachidonic acid-induced inflammation and indomethacin- induced gastric ulceration. Cell.

[B21] Morham SG, Langenbach R, Loftin CD, Tiano HF, Vouloumanos N, Jennette JC, Mahler JF, Kluckman KD, Ledford A, Lee CA (1995). Prostaglandin synthase 2 gene disruption causes severe renal pathology in the mouse. Cell.

[B22] Hall ED, Sullivan PG, Gibson TR, Pavel KM, Thompson BM, Scheff SW (2005). Spatial and temporal characteristics of neurodegeneration after controlled cortical impact in mice: more than a focal brain injury. J Neurotrauma.

[B23] Kelso ML, Wehner JM, Collins AC, Scheff SW, Pauly JR (2006). The pathophysiology of traumatic brain injury in alpha7 nicotinic cholinergic receptor knockout mice. Brain Res.

[B24] Dash PK, Mach SA, Moore AN (2000). Regional expression and role of cyclooxygenase-2 following experimental traumatic brain injury. J Neurotrauma.

[B25] Paxinos G, Franklin KBJ (2001). The mouse brain in stereotaxic coordinates.

[B26] Sullivan PG, Bruce-Keller AJ, Rabchevsky AG, Christakos S, Clair DK, Mattson MP, Scheff SW (1999). Exacerbation of damage and altered NF-kappaB activation in mice lacking tumor necrosis factor receptors after traumatic brain injury. J Neurosci.

[B27] Raghavendra Rao VL, Dogan A, Bowen KK, Dempsey RJ (2000). Traumatic brain injury leads to increased expression of peripheral-type benzodiazepine receptors, neuronal death, and activation of astrocytes and microglia in rat thalamus. Exp Neurol.

[B28] Stephenson DT, Schober DA, Smalstig EB, Mincy RE, Gehlert DR, Clemens JA (1995). Peripheral benzodiazepine receptors are colocalized with activated microglia following transient global forebrain ischemia in the rat. J Neurosci.

[B29] Choi SH, Langenbach R, Bosetti F (2008). Genetic deletion or pharmacological inhibition of cyclooxygenase-1 attenuate lipopolysaccharide-induced inflammatory response and brain injury. Faseb J.

[B30] Aid S, Langenbach R, Bosetti F (2008). Neuroinflammatory response to lipopolysaccharide is exacerbated in mice genetically deficient in cyclooxygenase-2. J Neuroinflammation.

[B31] Gopez JJ, Yue H, Vasudevan R, Malik AS, Fogelsanger LN, Lewis S, Panikashvili D, Shohami E, Jansen SA, Narayan RK (2005). Cyclooxygenase-2-specific inhibitor improves functional outcomes, provides neuroprotection, and reduces inflammation in a rat model of traumatic brain injury. Neurosurgery.

[B32] Cernak I, O'Connor C, Vink R (2002). Inhibition of cyclooxygenase 2 by nimesulide improves cognitive outcome more than motor outcome following diffuse traumatic brain injury in rats. Exp Brain Res.

[B33] Hall ED, Bryant YD, Cho W, Sullivan PG (2008). Evolution of post-traumatic neurodegeneration after controlled cortical impact traumatic brain injury in mice and rats as assessed by the de Olmos silver and fluorojade staining methods. J Neurotrauma.

[B34] Myers R, Manjil LG, Cullen BM, Price GW, Frackowiak RS, Cremer JE (1991). Macrophage and astrocyte populations in relation to [3H]PK 11195 binding in rat cerebral cortex following a local ischaemic lesion. J Cereb Blood Flow Metab.

[B35] Venneti S, Lopresti BJ, Wang G, Hamilton RL, Mathis CA, Klunk WE, Apte UM, Wiley CA (2009). PK11195 labels activated microglia in Alzheimer's disease and in vivo in a mouse model using PET. Neurobiol Aging.

[B36] Venneti S, Wang G, Wiley CA (2007). Activated macrophages in HIV encephalitis and a macaque model show increased [3H](R)-PK11195 binding in a PI3-kinase-dependent manner. Neurosci Lett.

[B37] Giusti L, Betti L, Giannaccini G, Mascia G, Bazzichi L, Lucacchini A (2004). [3H]PK11195 binding sites in human neutrophils: effect of fMLP stimulation and modulation in rheumatic diseases. Clin Biochem.

[B38] Clark RS, Kochanek PM, Schwarz MA, Schiding JK, Turner DS, Chen M, Carlos TM, Watkins SC (1996). Inducible nitric oxide synthase expression in cerebrovascular smooth muscle and neutrophils after traumatic brain injury in immature rats. Pediatr Res.

[B39] Clark RS, Schiding JK, Kaczorowski SL, Marion DW, Kochanek PM (1994). Neutrophil accumulation after traumatic brain injury in rats: comparison of weight drop and controlled cortical impact models. J Neurotrauma.

[B40] Holmin S, Mathiesen T, Shetye J, Biberfeld P (1995). Intracerebral inflammatory response to experimental brain contusion. Acta Neurochir (Wien).

[B41] Royo NC, Wahl F, Stutzmann JM (1999). Kinetics of polymorphonuclear neutrophil infiltration after a traumatic brain injury in rat. Neuroreport.

